# Seroprevalence of Epizootic Hemorrhagic Disease Virus in Guangdong Cattle Farms during 2013–2017, China

**DOI:** 10.3390/v15061263

**Published:** 2023-05-28

**Authors:** Min-Na Lv, Jian-Bo Zhu, Shen-Quan Liao, Zhen-Xing Yang, Xu-Hui Lin, Nan-Shan Qi, Qin-Ling Chen, Cai-Yan Wu, Juan Li, Hai-Ming Cai, Jian-Fei Zhang, Jun-Jing Hu, Wen-Wan Xiao, Xu Zhang, Ming-Fei Sun

**Affiliations:** 1 Key Laboratory of Livestock Disease Prevention of Guangdong Province, Key Laboratory of Avian Influenza and Other Major Poultry Diseases Prevention and Control, Ministry of Agriculture and Rural Affairs, Institute of Animal Health, Guangdong Academy of Agricultural Sciences, Guangzhou 510640, China; gdczmn@163.com (M.-N.L.); lsq6969@163.com (S.-Q.L.); linxuhui1988@163.com (X.-H.L.); nanshanqi@163.com (N.-S.Q.); chenqinling@126.com (Q.-L.C.); wucaiyan906@163.com (C.-Y.W.); lijuan413@126.com (J.L.); caihaiming@gdaas.cn (H.-M.C.); zhangjfei@tom.com (J.-F.Z.); sigrid0725@163.com (J.-J.H.); xww_942242920@163.com (W.-W.X.); 2 Yunnan Tropical and Subtropical Animal Virus Diseases Laboratory, Yunnan Animal Science and Veterinary Institute, Kunming 650224, China; zhujb70@126.com (J.-B.Z.); s300yn@163.com (Z.-X.Y.); 3 School of Life Science and Engineering, Foshan University, Foshan 528000, China

**Keywords:** epizootic hemorrhagic disease virus, cattle, seroprevalence, Guangdong province, *Culicoides*

## Abstract

Epizootic hemorrhagic disease (EHD) is an infectious viral disease caused by epizootic hemorrhagic disease virus (EHDV) and EHDV frequently circulates in wild and domestic ruminants. Sporadic outbreaks of EHD have caused thousands of deaths and stillbirths on cattle farms. However, not much is known about the circulating status of EHDV in Guangdong, southern China. To estimate the seroprevalence of EHDV in Guangdong province, 2886 cattle serum samples were collected from 2013 to 2017 and tested for antibodies against EHDV using a competitive ELISA. The overall seroprevalence of EHDV reached 57.87% and was highest in autumn (75.34%). A subset of positive samples were serotyped by a serum neutralization test, showing that EHDV serotypes 1 and 5–8 were circulating in Guangdong. In addition, EHDV prevalence always peaked in autumn, while eastern Guangdong had the highest EHDV seropositivity over the five-year period, displaying apparent temporal–spatial distribution of EHDV prevalence. A binary logistic model analysis indicated a significant association between cattle with BTV infections and seroprevalence of EHDV (OR = 1.70, *p* < 0.001). The co-infection of different serotypes of EHDV and BTV raises a high risk of potential genomic reassortment and is likely to pose a significant threat to cattle, thus urging more surveillance to monitor their circulating dynamics in China.

## 1. Introduction

Epizootic hemorrhagic disease (EHD), first described in white-tailed deer in New Jersey (USA), is an infectious viral disease circulating in wild and domestic ruminants [1]. The etiology is epizootic hemorrhagic disease virus (EHDV), a close relative of Bluetongue virus (BTV), belonging to genus *Orbivirus*, family *Sedoreoviridae* [2]. The transmission of EHDV is mediated by female competent *Culicoides* biting midges, involving seasonal circulation and the spatiotemporal abundance of certain *Culicoides* species [3,4,5]. 

EHD can cause a fulminant hemorrhagic syndrome in white-tailed deer that often results in high mortality rates [6]. Cattle infection of specific EHDV serotypes can develop abortion or stillbirth, a reduction in milk production, pyrexia, anorexia, and extensive mucosal and serosal bleeding, although similar infections of other wildlife species and livestock are frequently asymptomatic [7,8,9,10,11,12,13,14,15]. The morbidity and the case-fatality rate of EHDV infection in cattle differ between serotypes and can be up to 100% and 10%, respectively [6]. In addition to cattle and deer, sero-surveillance and viral nucleic acid detections have suggested that sheep, goat, yak, camel, and llama are potential hosts of EHDV [16]. However, commercially available vaccines or therapeutic measures that can prevent EHDV infection are still missing.

To date, EHDV has been classified into seven serotypes (EHDV-1, -2, and -4–8) according to VP2 protein phylogeny in addition to the two newly identified putative serotypes (EHDV-10 and EHDV YNDH/V079/2018) [17,18,19]. Among these serotypes, five (EHDV-1, -2, -6, -7, -8) were reported to be associated with clinical outbreaks of cattle infection [8,9,10,11,12,14,15,20,21]. The Ibaraki virus, a representative of serotype 2 (EHDV-2), is the causative agent of sporadic outbreaks in cattle and has led to thousands of deaths or stillbirths of cattle in Japan during the 1950s and 1990s [15,22,23]. More recently, clinical outbreaks associated with EHDV-2, EHDV-6, and EHDV-7 have been reported to cause cattle illness and death in more broad geographic areas, including the USA, Japan, Israel, Turkey, Réunion Island, the Mediterranean Basin, and South Africa [8,9,10,11,12,14]. At the end of 2021 and 2022, EHDV-8 outbreaks with clinical signs were reported in Tunisia and in southern Europe [21,24]. These recent clinical outbreaks of serotypes previously considered as non-pathogenic for cattle are characterized by expanded geographical distribution and increased pathogenicity, which highlighted the active global expansion of virulent EHDV.

As both are transmitted by hematophagous *Culicoides*, BTV and EHDV co-circulation have been reported in Trinidad, South America, Reunion Island, and French Guiana [25,26,27,28]. In addition to the shared global distribution pattern, BTV and EHDV infections sometimes cause similar clinical signs in cattle. However, EHDV infections were considered less important to animal agriculture than BTV and were largely neglected previously [6,29]. As more pathogenic EHDV strains (serotypes 1, 2, 6, 7, and 8) are rapidly expanding, they have attracted more attention from international authorities and have been included as notifiable multispecies diseases by the World Organization for Animal Health (Office International des Epizooties) [30]. Moreover, the molecular nature that has broadened the geographic distribution and pathogenic capacity of EHDV is scarcely known. Thus, molecular and serological surveillance are urgently needed to gain more information about the co-circulating status of different EHDV serotypes and co-infection of EHDV as well as BTV, which may inform the preparedness for possible epidemics of EHDV in the future.

In China, only EHDV-1, EHDV-5, EHDV-7, and a novel strain (EHDV/YNDH/V079/2018) have been isolated from a few sentinel calves in Yunnan province in the southwest part of China [18,31]. Serological evidence of EHDV-8 infection has also been reported in cattle and sheep [32]. However, the epidemiological status and distribution of different serotypes of EHDV in China are still largely unknown. This study aimed to determine the seroprevalence of EHDV in cattle and to characterize the temporal–spatial distribution of different EHDV strains in Guangdong province, southern China, a hotspot of *Culicoides* species. From 2013 to 2017, 2886 serum samples were collected and analyzed for antibodies against EHDV using a competitive enzyme-linked immunosorbent assay (c-ELISA). Moreover, positive samples were serotyped and the co-infection of EHDV and BTV was determined. Finally, a binary logistic model was performed to estimate the seropositive risk as a function of year, season, region, and BTV co-infection by year interaction.

## 2. Materials and Methods

### 2.1. Study Area

**Figure 1:**
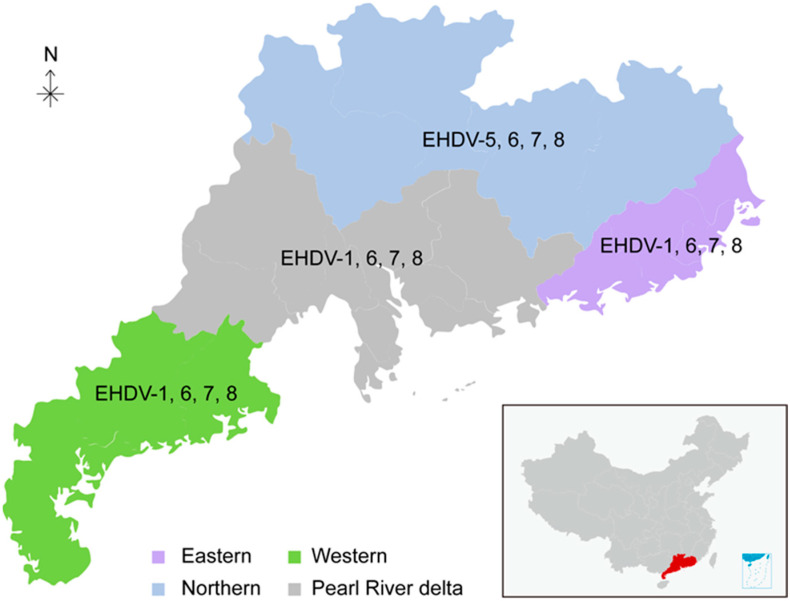
Geographic location of the sampling sites in Guangdong province. Eastern, Western, Northern, and Pearl River delta of Guangdong are shaded as indicated. The serotypes of EHDV in different regions are indicated.

Guangdong province, spanning between latitude 20°13’ and 25°31’, is located in southern China (179,770 km^2^) and has a tropical and subtropical humid climate with long summers. The monthly average temperature range is 11–37 °C. The relative humidity ranges from 70% to 90%. The annual rainfall is approximately 1800 mm (https://www.worldweatheronline.com/, accessed on 10 May 2022). This retrospective study recruited 30 beef cattle farms (8 in eastern Guangdong, 6 in western Guangdong, 8 in northern Guangdong, and 8 in the Pearl River delta) in 11 counties across Guangdong province (Figure 1).

### 2.2. Serum Samples

A total of 2886 blood samples from 30 beef cattle farms were collected from spring to autumn from 2013 to 2017. The age of sampled cattle ranged from 12 months to 18 months. All the blood samples were collected from different cattle throughout the study. Among the 2886 samples, 726 samples were collected during 2013, 916 during 2014, 419 during 2015, 482 during 2016, and 343 during 2017. Blood samples were collected from the jugular vein with tubes without anticoagulant and allowed to clot. The serum samples were separated from the whole blood by centrifugation and were stored at −20 °C until testing. The sera samples were aliquoted into several parts after centrifugation for different usage. In addition, the samples were grouped according to year, season, and locations.

### 2.3. Serology

Specific anti-EHDV antibodies in serum samples from cattle were tested using a commercial EHDV group-specific competitive ELISA kit (Kernel, Charlotte, NC, USA) based on VP7 protein. All tests were performed according to the manufacturer’s instructions and the cut-off values were set as suggested. Briefly, the negative controls and positive controls from the ELISA kit were set in duplicate on each plate. Optical densities at 450 nm (OD_450_) values were measured by a microplate reader (SpectraMax M2, Molecular Devices, San Jose, CA, USA). The results were determined by the percentage of inhibition (PI) = [(OD) of NC-OD of sample)/OD of NC] × 100. A mean PI value of the duplicate tests was used as the final result. When the OD_450_ > 0.6 and the PI of positive control ≥55%, the test could be considered valid. When the PI of a sample was more than 55%, it would be considered as positive and otherwise negative. 

For BTV, a competitive ELISA kit that is commercially available from IDEXX (Cat number: P00450-10) was used according to the manufacturer’s instructions. This kit is based on the competition between the sample to be tested and a monoclonal antibody, which binds to the VP7 protein, a major protein of the BTV. Briefly, 20 μL of un-diluted serum samples was used for testing. Two negative controls and one positive control were included on each plate. The OD_450_ was measured and recorded after incubation with a microplate reader. The assay was considered valid only if the OD mean of the negative control was ≥0.70 and ≤3.0 while S/N% of the positive control was ≤20%. For interpretation of the test, the sample was considered negative only if S/N% ≥ 80%. When the S/N% of a sample was ≤70%, it was considered positive. S/N% between 70% and 80% was considered suspicious and the test was repeated.

### 2.4. Serum Neutralization Test (SNT) 

A total of 76 randomly selected positive sera from 4 distinct areas (26 from eastern Guangdong, 11 from western Guangdong, 14 from northern Guangdong, and 25 from the Pearl River delta) were titrated to determine the neutralizing titer and the presence of serotype-specific EHDV antibodies by SNT. Each serum sample was tested against 6 EHDV serotypes: EHDV-1, -2, -5, -6, -7, and -8. All EHDV reference strains used here were provided by Yunnan Animal Science and Veterinary Institute, Kunming, China. These reference strains originated from Elizabeth Macarthur Agricultural Institute.

The development of a neutralizing antibody response was performed with BSR cells as described [33]. Briefly, sera (in duplicate) were prepared in MEM (Gibco, New York, NY, USA) with twofold dilutions starting at 1:2 (range of dilution: 2–256). Next, 50 μL of the diluted sera was incubated with 50 μL MEM containing 100 TCID_50_ of each EHDV serotype (EHDV-1, -2, -5, -6, -7, and -8) in microtiter plates for 1 h at 37 °C. Then, 2 × 10^4^ cells in 100 μL of MEM containing 10% fetal calf serum (Gibco) and 1% non-essential amino acid (Gibco) were added to each well. The plates were incubated at 37 °C for 7 days. The neutralizing titer was defined as the highest dilution allowing more than 50% neutralization of 100 TCID_50_. Titers were expressed as log_10_ of the reciprocal value of the endpoint serum dilution. Only samples with a dilution ≥10 that still can neutralize a specific EHDV serotype were considered as positive for that specific EHDV serotype (see Table S1). Otherwise, the tested sample was considered negative for a specific EHDV serotype.

### 2.5. Statistical Analysis

The epidemiologic data regarding year, season, and region were used as independent variables and the results of serological tests for EHDV were used as dependent variables. All independent variables were performed by cross-tabulation and descriptive statistics, such as frequency and percentage. Independent variables (Table 1, Table 2 and Table 3) were screened based on the response variable (ELISA) with a chi-square test (χ^2^). The epidemiologic data were applied to binary logistic models to assess the odds ratio (OR) for EHDV seropositivity. The differences were considered statistically significant with a *p*-value < 0.05 and statistically highly significant with a *p*-value < 0.001. All the above analyses were performed with SPSS software (version 15.0).

## 3. Results

### 3.1. Temporal–Spatial Distribution of EHDV Seroprevalence during 2013–2017

To estimate the circulating status of EHDV in southern China, 2886 cattle sera were collected from 30 sentinel farms in Guangdong province during 2013–2017 (Figure 1). With c-ELISA, 1670 out of the 2886 samples were tested positive for antibodies against EHDV, with an overall seropositive rate of 57.87% (95% CI: 56.06–59.67%) (Table 1). The EHDV seropositive rate was the highest in 2016 and reached 81.12% (95% CI: 77.61–84.63%) (Table 1). The seropositive rate was the lowest in 2013 (43.53% (95% CI: 39.91–47.14%)), and the seropositive rate was 57.86% in 2014, 59.67% in 2015, and 53.35% in 2018. The five-year overall positive rates for the Pearl River delta, eastern, western, and northern regions were 54.81% (95% CI: 52.09–57.53%), 75.21% (95% CI: 71.70–78.72%), 49.75 (95% CI: 44.88–54.63%), and 53.07% (95% CI: 49.07–57.06%), respectively (Table 1). The EHDV seropositive rate of eastern Guangdong is significantly higher than other regions over the five-year period.

Notably, the annual EHDV seropositive rate in the eastern part of Guangdong was always significantly higher than in other parts over all five years (Table 2). Strikingly, the EHDV seropositive rate of eastern Guangdong in 2016 reached 94.55% (95% CI: 90.23–98.86%) (Table 2). Temporally, the annual seroprevalence of EHDV in cattle increased by season from spring to autumn (Table 1 and Table 3). The EHDV seropositive rate in spring over five years was only 33.33% (95% CI: 30.16–36.51%), which is much lower than autumn, which has a seropositive rate of 75.34% (95% CI: 72.71–77.96%) (Table 1). Moreover, the seroprevalence rates of BTV and EHDV infection were 64.38% (95% CI: 62.63–66.13%) and 57.87% (95% CI: 56.06–59.67%), respectively (Table 4). Strikingly, the seropositive rate of EHDV and BTV co-infection reached 40.23% (95% CI: 38.44–42.02%) (Table 4).

### 3.2. Circulating EHDV Serotypes in Guangdong

A total of 76 positive sera were randomly selected and titrated for the presence of six serotype-specific EHDV antibodies (EHDV-1, -2, -5, -6, -7, and -8). Six samples could not be determined among the above samples, indicating that other serotypes may exist (Table 5 and Table S1). Five serotypes, EHDV-1, -5, -6, -7, and -8, were detected, while EHDV-2 was not observed (Table 5 and Table S1). Among the detected serotypes, the EHDV-7 seropositive rate (53.95%, 95% CI: 42.48–65.41%) is the highest, followed by EHDV-6, EHDV-8, EHDV-1, and EHDV-5 (Table 5). Notably, co-infections of EHDV-1/7, EHDV-1/6/7, EHDV-1/6/8, EHDV-1/6/7/8, EHDV-5/8, EHDV-6/7, EHDV-6/8, EHDV-7/8, and EHDV-6/7/8 were all observed (Table S2). Although EHDV-6, -7, and -8 were detected in all four studied regions of Guangdong, EHDV-7 and EHDV-8 seroprevalence were mainly in eastern Guangdong, while EHDV-6 and EHDV-7 seroprevalence were predominantly in the Pearl River delta (Table 5). EHDV-5 seroprevalence was only found in northern Guangdong and EHDV-1 seroprevalence was absent in northern Guangdong (Table 5). These results show the slight geographic distribution bias of different EHDV serotypes (Figure 1).

### 3.3. Associated Factors of EHDV Infection

We performed binary logistic regression models to describe associated factors of seroprevalence for EHDV. The model included year, season, region, and BTV co-infection (Table 1), the interaction between region and year (Table 2), and the interaction between season and year (Table 3). These analyses were based on 2886 test results. In the year-adjusted model (Table 1), the odds of seropositivity for EHDV varied from year to year. In 2016, a significant difference existed compared with the other years (OR = 5.58, *p* < 0.001). Furthermore, the odds of seropositivity in eastern Guangdong were greater than other regions from 2013 to 2017 (Table 1 and Table 2). A significant difference was also observed between summer and spring (OR = 3.07, *p* < 0.001) and between autumn and spring (OR = 6.11, *p* < 0.001) (Table 1 and Table 3). Cattle with BTV infections were significantly associated with the seroprevalence of EHDV (OR = 1.70, *p* < 0.001) (Table 1).

## 4. Discussion

EHDV is an important pathogen in white-tailed deer and cattle; thus, EHD has been included in the OIE notifiable diseases list [30]. The presented retrospective seroprevalence study showed that at least five EHDV serotypes, including EHDV-1, -6, -7, and -8, which have been associated with clinical outbreaks in cattle farms, were circulating in Guangdong province, southern China. EHDV-6 infection in China was identified for the first time. The five-year overall EHDV seropositive rate of 2886 cattle was as high as 57.87% (95% CI: 56.06–59.67%) (Table 1). Moreover, both co-infections of different EHDV serotypes and co-infection of BTV as well as EHDV were observed. A significant association between exposure to EHDV and BTV was demonstrated in logistic regression analyses (OR = 1.70, *p* < 0.001) (Table 1).

The high overall EHDV seropositivity (57.87%) suggested that EHDV infection in cattle is widely spread in different regions of Guangdong, and the seropositive rate is significantly higher than other regions of China [16]. Previous studies indicated that the circulation dynamics of EHDV depend on the vector population abundance and vitality, which are largely influenced by climatic factors such as temperature, precipitation, wind, and humidity [34,35]. Guangdong province has a typical subtropical climate that is characterized by high temperature over the whole year, abundant rainfall, and high humidity, thus making it a preferred habitat of *Culicoides* species [36]. Therefore, the observed high overall prevalence may be partially mediated by the rich vitality and abundance of the *Culicoides* population compared with other parts of China, which is also supported by the fact that the incidence of EHDV infections increases from northern to southwestern China (from 0% to more than 50%) as the latitude decreases [16]. Annually, the seroprevalence of EHDV was highest in 2016 (81.12%) and a significant difference was observed compared with the other years (OR = 5.58, *p* < 0.001). Among the four investigated regions, eastern Guangdong has the highest overall EHDV seropositive rate of 75.21% and peaked in 2016 with a striking seropositive rate of 94.55%. The annual seropositive rate of EHDV in eastern Guangdong was always higher than other regions, and EHDV seroprevalence consistently peaked in autumn and was lowest in the spring over the investigated five-year period (Table 2 and Table 3). In addition, regression models also suggested statistically significant spatiotemporal variations in EHDV seroprevalence in Guangdong. Although it is reasonable that these temporal–spatial variations likely originated from vector vitality and abundance, the complex interactions between *Culicoides* and environmental as well as climatic factors have not been elucidated in Guangdong province. Moreover, the *Culicoides* species can vary significantly between different habitat regimes, urging the detailed characterization of the *Culicoides* composition and modeling the distribution of midges in Guangdong, which may benefit the efficient control of orbivirus transmission in economic animals.

The co-circulation of BTV and EHDV has been reported in several countries and regions [27,28,29]. Kedmi et al. compared the prevalence of both viruses in dairy cattle in Israel and found that the two viruses have different patterns of distribution, that of EHDV being affected by wind while that of BTV by herd immunity [29]. In our study, we could show that a significant association exists between BTV exposure and EHDV prevalence. It is hard to directly compare our study with that of Kedmi, since these two studies were conducted in different geographic and climate conditions. In addition, the vector abundance and composition that can largely influence EHDV as well as BTV infection of these two different areas may differ significantly. 

Genome segment reassortment can shape novel genotypes of BTV, which has also been observed in EHDV [37]. It remains elusive whether the reassortment contributes to the increasing virulence of EHDV. However, it has been reported that inner- or inter-serotypes genome segment reassortment of EHDV shaped novel topotypes with extended geographical distribution and host range [20,38,39]. The current study showed that the co-infection of different EHDV serotypes and co-infection of EHDV and BTV rates are high, which should catch the attention of local authorities. Notably, some EHDV strains that can cause clinical symptoms in cattle herds are circulating in Guangdong. Thus, active molecular surveillance, virus isolation, and genome sequencing of EHDV should be conducted in the future to reveal more details about the epidemiological consequences of EHDV genome segment reassortment.

Indeed, our study has some limitations. The serotyping results from 76 samples showed that several serotypes of EHDV, including serotypes 1, 5, 6, 7, and 8, were circulating in Guangdong; in addition, the seropositive rate is relatively high in Guangdong. Although EHDV-6 was identified serologically, more molecular characterizations are needed to distinguish EHDV-6 and EHDV-8 since it has been reported that cross-reactions between these two serotypes are high [21]. Furthermore, the genomic sequences of EHDV serotypes 1, 5, 6, 7, and 8 were not determined in this study. It is not known if these EHDV serotypes are identical to those circulating in eastern Asia. Thus, their evolutionary route is still mysterious, which urges more virological and molecular investigations to be carried out in the future. In addition, our study is retrospective and the information about the clinical symptoms of the cattle infected by EHDV serotypes 1, 6, 7, and 8 or their co-infection was missing. Thus, a more detailed epidemiological study should be performed in the future to gain more insight into the clinical significance of EHDV in cattle in Guangdong.

## 5. Conclusions

In conclusion, the current study presented the circulating dynamics of EHDV and its co-infection with BTV in cattle in Guangdong Province, southern China, over a five-year period. Factors associated with EHDV infection in cattle herds involved season, region, and co-infection with BTV. Moreover, serological results imply that EHDV-6 infection may undergo genomic reassortment in Guangdong, China. The high co-infection rate of BTV and EHDV as well as different serotypes of EHDV emphasized the importance of extensive monitoring of EHD epidemic status in China.

## Figures and Tables

**Table 1:** Univariable logistic regression analysis of factors associated with seroprevalence of EHDV in cattle in Guangdong.

Variables	Category	Number Tested (Seroprevalence %)	95% CI of Seroprevalence %	Odds Ratio	*p*-Value
Year	2013	726 (43.53)	39.91–47.14	Referent	—
	2014	916 (57.86)	54.66–61.06	1.78 (1.46–2.17)	<0.001
	2015	419 (59.67)	54.95–64.38	1.92 (1.50–2.45)	<0.001
	2016	482 (81.12)	77.61–84.63	5.58 (4.25–7.31)	<0.001
	2017	343 (53.35)	48.05–58.66	1.48 (1.15–1.92)	0.003
Season	Spring	852 (33.33)	30.16–36.51	Referent	—
	Summer	992 (60.58)	57.54–63.63	3.07 (2.54–3.72)	<0.001
	Autumn	1042 (75.34)	72.71–77.96	6.11 (5.0–7.46)	<0.001
Region	Eastern	585 (75.21)	71.70–78.72	3.06 (2.34–4.01)	<0.001
	Western	408 (49.75)	44.88–54.63	Referent	—
	Northern	603 (53.07)	49.07–57.06	1.14 (0.89–1.47)	0.301
	Pearl River delta	1290 (54.81)	52.09–57.53	1.23 (0.98–1.53)	0.075
Overall	—	2886 (57.87)	56.06–59.67	—	—
BTV infection	No	1028 (49.51)	46.45–52.58	Referent	—
	Yes	1858 (62.49)	60.28–64.69	1.70 (1.46–1.98)	<0.001

**Table 2:** Odds ratios (OR) of seropositivity of EHDV in the studied regions of Guangdong over 2013–2017.

Year	Region	Number Tested (Seroprevalence %)	95% CI of Seroprevalence %	Odds Ratio (95% CI)	*p*-Value
2013	Eastern	130 (58.46)	49.88–67.05	4.62 (2.54–8.43)	<0.001
	Western	90 (23.33)	14.43–32.24	Referent	—
	Northern	146 (38.36)	30.38–46.34	2.04 (1.13–3.69)	0.017
	Pearl River delta	360 (45.28)	40.11–50.44	2.72 (1.60–4.62)	<0.001
2014	Eastern	180 (76.67)	70.43–82.90	3.46 (2.09–5.73)	<0.001
	Western	115 (48.70)	39.42–57.97	Referent	—
	Northern	206 (52.43)	45.55–59.30	1.16 (0.74–1.83)	0.521
	Pearl River delta	415 (54.94)	50.13–59.75	1.29 (0.85–1.94)	0.235
2015	Eastern	80 (78.75)	69.59–87.91	3.23 (1.60–6.55)	0.001
	Western	73 (53.42)	41.71–65.14	Referent	—
	Northern	90 (51.11)	40.58–61.64	0.91 (0.49–1.69)	0.769
	Pearl River delta	176 (57.95)	50.59–65.32	1.20 (0.69–2.08)	0.511
2016	Eastern	110 (94.55)	90.23–98.86	4.13 (1.52–11.19)	0.003
	Western	78 (80.77)	71.83–89.71	Referent	—
	Northern	94 (81.91)	73.99–89.84	1.08 (0.50–2.33)	0.848
	Pearl River delta	200 (73.50)	67.33–79.67	0.66 (0.35–1.26)	0.205
2017	Eastern	85 (69.41)	59.41–79.41	2.65 (1.30–5.41)	0.007
	Western	52 (46.15)	32.14–60.17	Referent	—
	Northern	67 (49.25)	36.97–61.54	1.13 (0.55–2.34)	0.737
	Pearl River delta	139 (48.20)	39.79–56.61	1.09 (0.57–2.06)	0.801

**Table 3:** Odds ratios (OR) of seropositivity of EHDV by season over 2013–2017.

Year	Region	Number Tested (Seroprevalence %)	95% CI of Seroprevalence %	Odds Ratio (95% CI)	*p*-Value
2013	Spring	212 (22.64)	16.96–28.32	Referent	—
	Summer	240 (48.33)	41.97–54.70	3.20 (2.12–4.81)	<0.001
	Autumn	274 (55.47)	49.55–61.40	4.26 (2.85–6.35)	<0.001
2014	Spring	275 (29.82)	24.38–35.26	Referent	—
	Summer	300 (57.33)	51.70–62.96	3.16 (2.24–4.47)	<0.001
	Autumn	341 (80.94)	76.75–85.13	10.0 (6.88–14.53)	<0.001
2015	Spring	115 (28.70)	20.30–37.09	Referent	—
	Summer	162 (62.96)	55.45–70.48	4.22 (2.53–7.07)	<0.001
	Autumn	142 (80.99)	74.45–87.52	10.58 (5.91–18.94)	<0.001
2016	Spring	150 (64.67)	56.93–72.40	Referent	—
	Summer	170 (85.88)	80.59–91.17	3.32 (1.93–5.74)	<0.001
	Autumn	162 (91.36)	86.98–95.73	5.78 (3.04–10.98)	<0.001
2017	Spring	100 (24.0)	15.48–32.52	Referent	—
	Summer	120 (54.17)	45.12–63.21	3.74 (2.09–6.70)	<0.001
	Autumn	123 (76.42)	68.82–84.03	10.26 (5.52–19.07)	<0.001

**Table 4:** EHDV, BTV, and their co-infection seropositive rate.

Type	Number Tested (Seroprevalence %)	95% CI of Seroprevalence %
EHDV	1670 (57.87)	56.06–59.67
BTV	1858 (64.38)	62.63–66.13
EHDV + BTV	1161 (40.23)	38.44–42.02
Negative	519 (17.98)	16.58–19.39

**Table 5:** Serotyping of EHDV in tested cattle, Guangdong, 2013–2017.

	EHDV-1	EHDV-2	EHDV-5	EHDV-6	EHDV-7	EHDV-8	Failed to Determine
Eastern (*n* = 26)	6	0	0	6	11	9	1
Western (*n* = 11)	2	0	0	2	5	1	3
Northern (*n* = 14)	0	0	5	2	4	4	1
Pearl river delta (*n* = 25)	6	0	0	10	21	4	1
Ratio (%)	18.42	-	6.58	26.32	53.95	23.68	—
95% CI of ratios	9.50–27.34	-	0.88–12.28	16.19–36.45	42.48–65.41	13.90–33.46	—

## Data Availability

All data are available upon request.

## Supplementary Materials

The following supporting information can be downloaded at: https://www.mdpi.com/article/10.3390/v15061263/s1, Table S1: Neutralizing antibody titers of EHDV serotypes 1, 5, 6, 7, and 8 of tested samples (*n* = 76), Guangdong, 2013–2017; Table S2: Serotyping details of EHDV in tested cattle, Guangdong, 2013–2017.
