# Supplementary material for: Seroprevalence of Epizootic Hemorrhagic Disease Virus in Guangdong Cattle Farms during 2013–2017, China
Source: Viruses. 2023 May 28;15(6):1263. doi: 10.3390/v15061263 (PMC10303234; doi:10.3390/v15061263)
Supplement: Supplementary file 1 [file viruses-15-01263-s001.zip › viruses-2405503-supplementary.pdf]

Supplemental Table S1. Neutralizing antibody titers of EHDV serotype 1, 5, 6, 7, and 8 of tested samples (n=76), Guangdong, 2013-2017.

Titer	EHDV Serotypes				
	EHDV-1 (n=14)	EHDV-5 (n=5)	EHDV-6 (n=20)	EHDV-7 (n=41)	EHDV-8 (n=18)
≥10, < 20	2	0	2	10	1
≥20, < 40	3	1	10	7	1
≥40	9	4	8	24	16

Supplemental Table S2. Serotyping details of EHDV in tested cattle, Guangdong, 2013-2017.

Serotypes	Regions			
	East (n=25)	West (n=8)	North (n=13)	Pearl river delta (n=24)
EHDV-1	4	2		
EHDV-5			4	
EHDV-6	4		1	2
EHDV-7	7	3	4	8
EHDV-8	6	1	2	
EHDV-1+7	1			4
EHDV-1+6+7				1
EHDV-1+6+8				1
EHDV-1+6+7+8	1			
EHDV-5+8			1	
EHDV-6+7		2		5
EHDV-6+8			1	
EHDV-7+8	1			2
EHDV-6+7+8	1			1
